# Posterior approach achieves more accurate replication of the posterior horn than anterior approach in transtibial pull-out repair of medial meniscus posterior root tear

**DOI:** 10.1186/s43019-026-00302-y

**Published:** 2026-02-27

**Authors:** Dong-Wook Son

**Affiliations:** Department of Orthopedic Surgery, Kangbuk Samsung Hospital, Sungkyunkwan University School of Medicine, 29 Saemunan-Ro, Jongno-Gu, Seoul, 03181 Republic of Korea

**Keywords:** Medial meniscus, Posterior root tear, Tunnel position, Accuracy, Outcomes

## Abstract

**Purpose:**

We aimed to compare the anterior and posterior approach for transtibial pull-out repair of medial meniscal posterior root tear (MMPRT) in terms of tibial tunnel positioning of the posterior horn, healing status, medial meniscal extrusion (MME), medial joint space (MJS) narrowing, and clinical outcomes.

**Methods:**

This retrospective study included patients who underwent arthroscopic transtibial pull-out repair for MMPRT between May 2019 and June 2023. Tibial tunnel positioning was assessed postoperatively using computed tomography. The healing status was evaluated using magnetic resonance imaging (MRI) at the 1-year follow-up visit. Pre- and postoperative MME and MJS widths were measured using MRI and weight-bearing radiography, respectively. Clinical outcomes were assessed preoperatively and at the 2-year follow-up using the International Knee Documentation Committee subjective score, Lysholm score, Knee Injury and Osteoarthritis Outcome Score, and Tegner activity scale.

**Results:**

A total of 77 patients were initially evaluated for eligibility, of whom 23 were excluded. A total of 54 patients were analyzed (26 anterior approach (AA), 28 posterior approach (PA)). The PA group demonstrated significantly more accurate tibial tunnel positioning compared with the AA group (mean absolute distance: 2.8 ± 2.0 mm versus 4.9 ± 3.2 mm, *p* = 0.001). MRI at follow-up demonstrated that complete or partial healing was achieved in 88.9% of cases, with no significant difference between groups (*p* = 0.413). Overall, MME increased from 3.0 ± 0.9 mm to 4.0 ± 1.6 mm (*p* = 0.022) and MJS decreased from 3.5 ± 1.2 mm to 3.1 ± 1.3 mm (*p* = 0.001), without intergroup differences. All clinical scores improved significantly from baseline, but no significant differences were observed between approaches at final follow-up.

**Conclusion:**

The PA group achieved more accurate replication of the posterior horn insertion than the AA group; however, no definitive advantages were observed in short-term clinical or radiographic outcomes.

*Level of evidence*: III, retrospective comparative cohort study.

## Introduction

Medial meniscal posterior root tears (MMPRTs) disrupt the circumferential hoop stress, leading to pathological extrusion and increased medial tibiofemoral pressure, biomechanically comparable to total medial meniscectomy [1]. When managed nonoperatively, MMPRTs can result in rapid osteoarthritis (OA) progression, and medial joint space (MJS) narrowing [2]. Transtibial pull-out repair is the standard treatment, showing favorable mid- to long-term outcomes by limiting extrusion during flexion [3–5]. However, it does not completely prevent osteoarthritis progression, and postoperative medial meniscus extrusion (MME) frequently persists, especially within the first year [6–10].

The success of pull-out repair depends on patient factors, surgical timing, and healing capacity [11–13]. Accurate tibial tunnel placement is critical, as anatomical positioning restores native joint mechanics and reduces MME, while nonanatomical placement may compromise meniscal healing and outcomes [14, 15].

Although anatomical tunnel placement has been associated with improved meniscal healing and reduced MME [15, 16], the relationship between tibial tunnel position and surgical approach remains poorly understood in clinical studies. Posterior knee arthroscopy is considered safe and provides superior visualization in all-inside repair of posterior meniscal tears, with demonstrated clinical benefits [17]. This approach, however, is not widely adopted, and reports of its use are limited [18]. MMPRTs are posteriorly located and can be visualized and managed with a posterior portal technique, yet few studies have evaluated the effectiveness of posterior surgical repair.

This study aims to compare the differences between the anterior approach (AA) and posterior approach (PA) for MMPRT pull-out repair, specifically in terms of tibial tunnel placement accuracy, MJS narrowing, MME, and clinical outcomes. We hypothesized that the PA would enable more accurate tibial tunnel placement and result in reduced postoperative MME and MJS narrowing, along with improved clinical outcomes, compared with the standard AA technique.

## Methods

This study was approved by our Institutional Review Board. Data were retrospectively collected from patients who underwent surgical treatment for MMPRTs at our institution between May 2019 and June 2023. The operative indications for transtibial pull-out repair included a hip–knee–ankle angle of varus ≤ 5°, Kellgren–Lawrence (K–L) grade of 0–2, and a mild cartilage lesion (Outerbridge grade I or II) as confirmed by preoperative radiographs and MRI. Patients were included if they (1) experienced persistent knee pain with a diagnosis of MMPRT confirmed by MRI, (2) underwent arthroscopic pull-out fixation, and (3) had clinical outcomes assessed with a minimum follow-up of 2 years. Patients were excluded if they (1) lacked sufficient clinical outcome data or postoperative CT imaging, (2) had not undergone MRI at a minimum follow-up of 1 year, (3) underwent concomitant high tibial osteotomy and/or cartilage procedure, (4) had a history of prior knee surgery, or (5) received combined treatment for MMPRT repair. No patients were excluded from surgery on the basis of age, body mass index (BMI), symptom duration, chronicity of the tear, or activity level.

Demographic data, including age, sex, height, weight, body mass index (BMI), and interval from injury to surgery, were recorded for each patient. Clinical scores were assessed through patient interviews. Clinical evaluations were conducted preoperatively, at 6 and 12 months postoperatively, and annually thereafter. Patient identification was performed using the medical records and a prospectively maintained database. Demographic and clinical data were obtained through a retrospective review of the medical records. Postoperative CT and MRI were routinely performed to assess tibial tunnel positioning and meniscal healing status.

### Surgical procedure and rehabilitation

All MMPRTs were initially repaired using the AA through the anteromedial portal. Considering that posterior knee arthroscopy is a safe technique that enhances visualization and supports favorable outcomes in all-inside posterior meniscal repair, the PA was subsequently adopted for MMPRT repair and tunnel drilling. This technique, employing transseptal and posteromedial portals, was introduced in June 2021. Following further refinement, the PA has been the standard method for all MMPRT repairs since September 2021. There was no change in surgical indications or postoperative rehabilitation protocols during the study period.

**Fig. 1:**
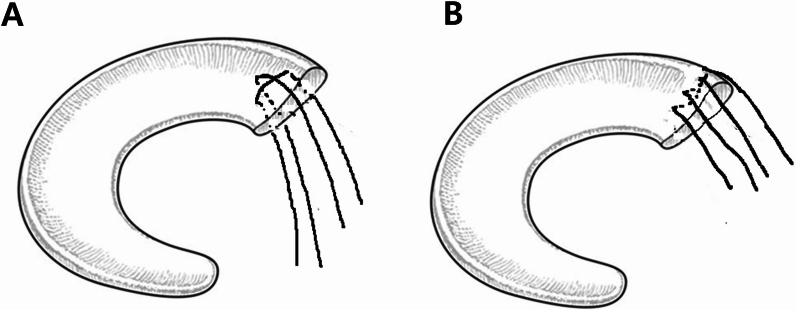
Schematic illustration of four suture ends from two strands placed using a suture hook at the posterior horn of the medial meniscus: **A** modified Mason–Allen technique, **B** two simple vertical sutures placed through the posteromedial portal

MMPRT was initially confirmed via arthroscopic examination (Fig. 1A, D). The torn site was debrided using a shaver, and a curette was used to create a bone bed at the native MM root insertion site. For the initial repair, a crescent-shaped suture hook (Linvatec, Largo, FL) loaded with a #1 polydioxanone (PDS) suture (Ethicon, Somerville, NJ) was used. The PDS suture was subsequently replaced with a no. 2 Ethibond suture (Ethicon, Somerville, NJ) using the shuttle-relay technique.

**Fig. 2:**
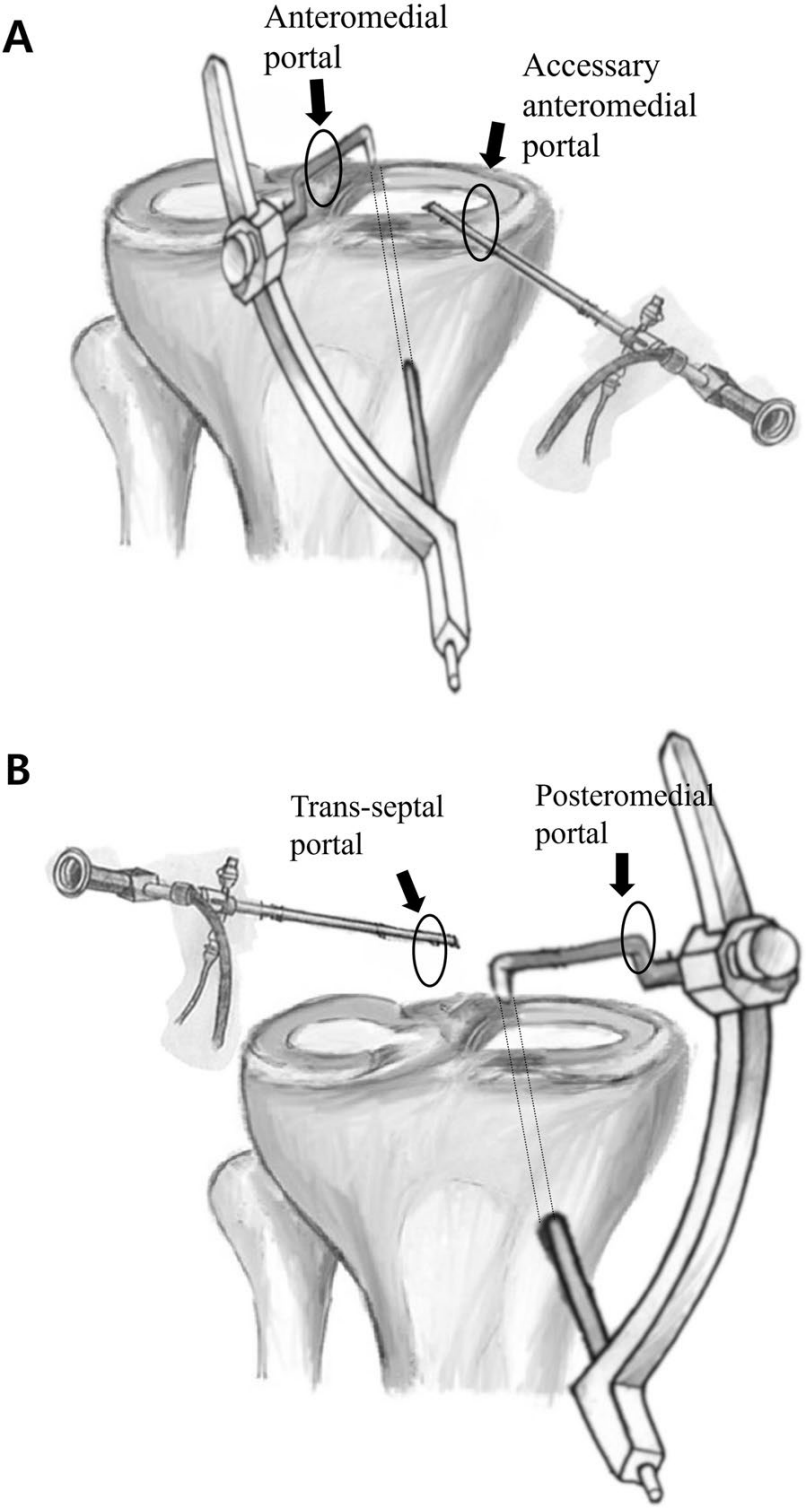
Schematic illustration of anterior and posterior approach techniques. **A** In the anterior approach, the arthroscope was inserted through an accessory anteromedial portal, and the ACL guide was introduced via the standard anteromedial portal. **B** In the posterior approach, the arthroscope was inserted through the transseptal portal, and the ACL guide was introduced via the posteromedial portal. The tibial tunnel was placed at the anteromedial region of the tibial tuberosity

**Fig. 3:**
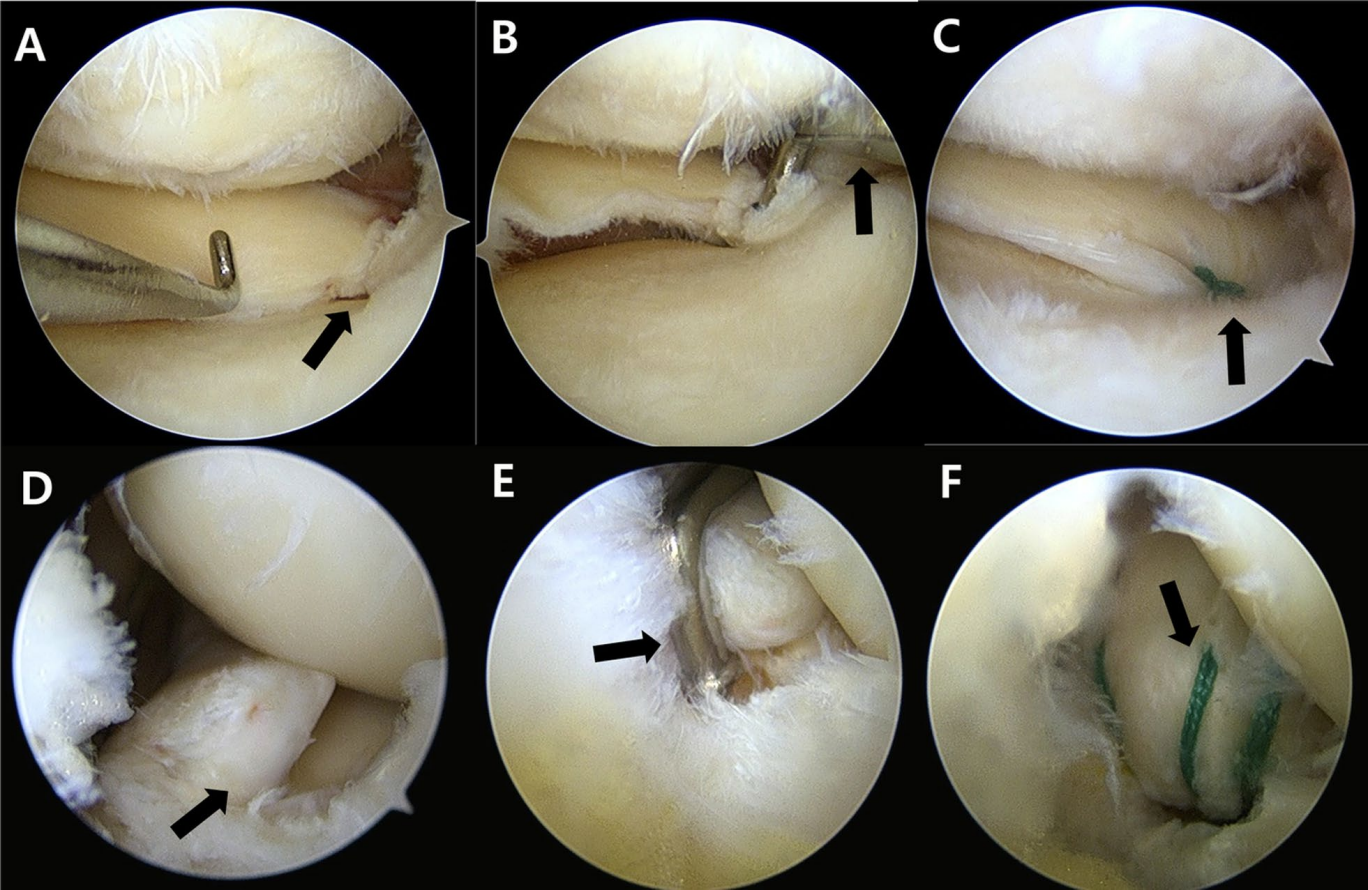
Two patients who underwent transtibial pull-out suture repair for medial meniscus posterior root tear (MMPRT) via the anterior and posterior approaches. **A** A 56-year-old woman with an MMPRT visualized through the anteromedial portal of the right knee (black arrow). **B** Arthroscopic view of a tibial tunnel guide (black arrow), designed for ACL reconstruction, introduced through the anteromedial portal and visualized via an additional accessory anteromedial portal. **C** MMPRT repair was performed using the modified Mason–Allen technique (black arrow), followed by transtibial pull-out suturing. **D** A 58-year-old woman with an MMPRT, visualized through the transseptal portal of the right knee. (black arrow). **E** Arthroscopic view of a tibial tunnel guide (black arrow), designed for ACL reconstruction, introduced through the posteromedial portal and visualized via the transseptal portal. **F** Two simple vertical sutures were placed using a suture hook at the posterior horn of the medial meniscus through the posteromedial portal (black arrow)

In the AA group, the modified Mason–Allen technique was applied (Fig. 2A). A horizontal mattress suture was placed approximately 5 mm medial to the torn margin of the meniscus, followed by a simple vertical suture overlaying the horizontal stitch. A tibial tunnel guide (Linvatec, Largo, FL) designed for anterior cruciate ligament (ACL) reconstruction was introduced through the anteromedial portal and visualized via an accessory anteromedial portal (Figs. 1B, 3A). A Kirschner wire was advanced through the tibial guide, and its tip was positioned at the far-lateral portion of the bone bed, just medial to the PCL footprint.

In the PA group, two simple vertical sutures were placed in the posterior portion of the meniscus using a suture hook inserted through the posteromedial portal (Fig. 2B). This technique was selected owing to the difficulty of performing the modified Mason–Allen technique through the posteromedial portal. However, adequate fixation strength was achieved by suturing the thick posterior meniscal tissue using this approach. The same tibial tunnel guide, designed for ACL reconstruction, was then introduced through the posteromedial portal and visualized via a transseptal portal with a 70° arthroscope. The tip of the ACL guide was positioned at the same site as in the AA (Figs. 1E, 3B).

Following tunnel preparation, which varied between the AA and PA groups, a 2.4-mm guide pin was inserted at a 45−65° angle (AA group: 45−55° and PA group: 55−65°) relative to the articular surface. This tunnel was then overdrilled using a 5-mm cannulated drill. The sutures were then retrieved through the tibial tunnel and secured to a 4.0-mm cancellous screw (DePuy Synthes, Raynham, MA, USA), which was placed 10 mm distal to the extra-articular tunnel outlet. Tibial fixation was performed with the knee flexed between 20° and 45°, under an initial tension of 20–30 N using a tensiometer.

Postoperative rehabilitation was conducted according to a standardized protocol for all patients. A hinged knee brace was applied postoperatively with the range of motion initially limited to 90°. Range-of-motion exercises began immediately and were gradually advanced, allowing flexion up to 90° by 3 weeks and 120° by 6 weeks. Full range of motion was permitted at 3 months. Toe-touch weight-bearing with crutches was allowed immediately and maintained for 3 weeks. Partial weight-bearing began at 3 weeks, followed by full weight-bearing and closed kinetic chain strengthening at 6 weeks. Daily activities resumed at 3 months, with running and return to sports allowed at 4 and 6 months, respectively.

### Three-dimensional computed tomography-based assessments

Postoperative computed tomography (CT) scans were performed 1 day after surgery using three-dimensional (3D)-reconstructed CT images. The tibial surface was reconstructed in 3D and evaluated using a rectangular grid system.

**Fig. 4:**
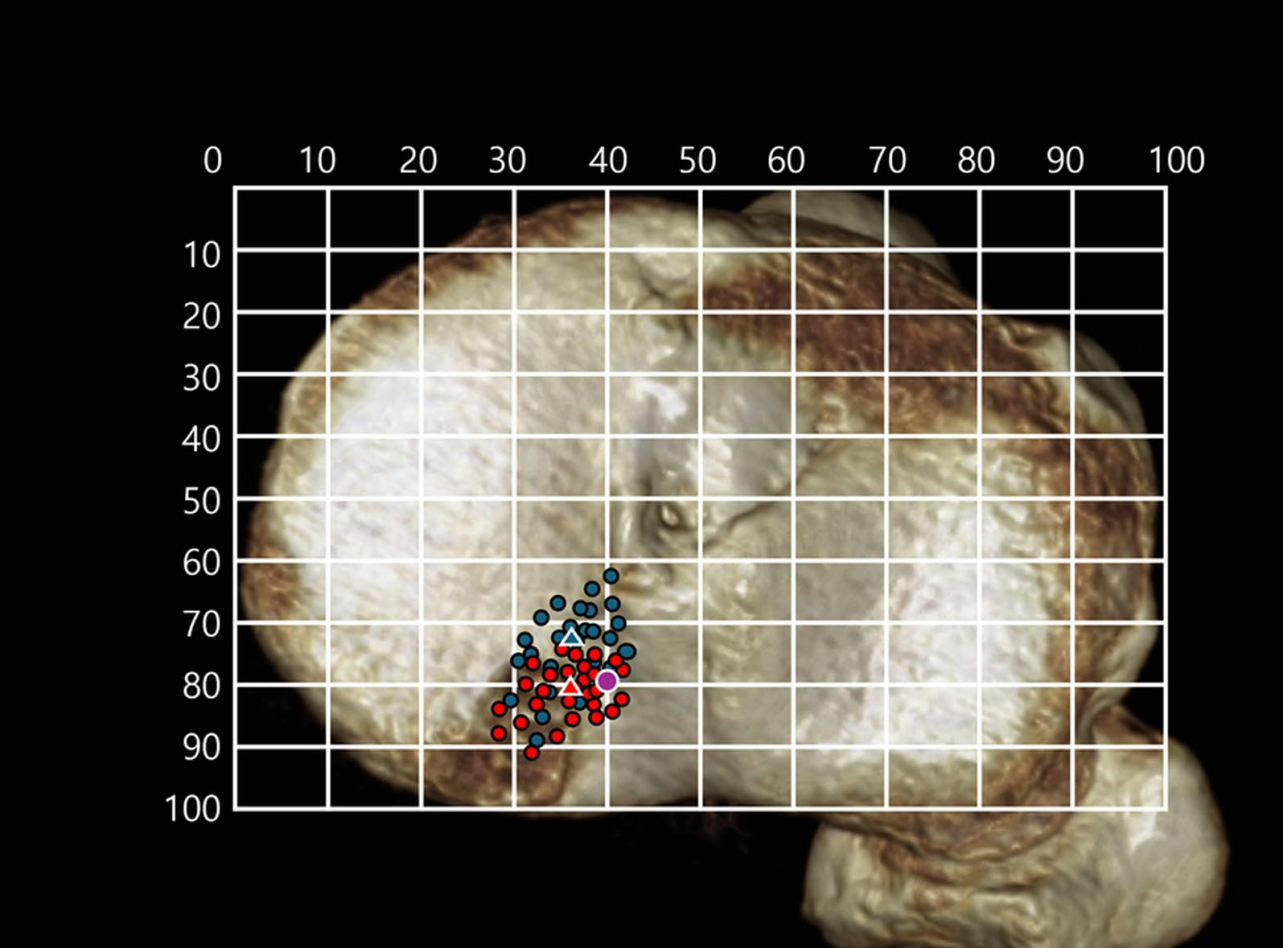
Tibial tunnel positions. Tibial tunnel locations are illustrated on a three-dimensional computed tomography-based tibial surface. Blue triangles indicate the tunnel positions in patients who underwent the anterior approach. Red triangles indicate those in the posterior approach group. The purple circle denotes the anatomic center of the medial meniscus posterior root attachment

The tibial axial rotation for anatomical positioning was standardized by aligning the posterior borders of the medial and lateral tibial plateaus horizontally in the axial view (Fig. 4). The anatomical attachment site of the MM posterior root was identified as the center of a circle delineated by three anatomical landmarks: the anterior margin of the tibial attachment of the PCL, the lateral border of the medial tibial plateau, and the posterior margin of the medial tibial eminence [19]. The tibial tunnel position was defined as the center of the tibial tunnel aperture.

The distance between the anatomical attachment site and the tibial tunnel position was measured in the posterior and lateral directions. The direct distance between the two points was calculated using the Pythagorean theorem, whereby the direct distance was defined as the square root of the sum of the squares of the posterior and lateral distances: (direct distance)^2^ = (posterior distance)^2^ + (lateral distance)^2^ [19].

### Radiographic assessments

The hip–knee–ankle angle was measured on full-length weight-bearing radiographs to evaluate the preoperative lower limb alignment. The Rosenberg 45° posteroanterior view was used to assess the K–L grade and OA severity on the basis of JSW [20].

Medial JSW was measured manually using a picture archiving and communication system (PACS). The measurement points were identified as the bright subchondral cortical bone lines of the tibial plateau and femoral condyle at the medial joint margins. The narrowest point along a vertical line between the medial femoral condyle and the medial tibial plateau was identified. The shortest distance between these points was recorded as the medial JSW [20].

Radiological assessments were performed preoperatively; at 6 weeks, 3, 6, and 12 months postoperatively and annually thereafter. Preoperative K–L grades and medial JSW were compared with the 2-year postoperative values within each group, and the outcomes were then compared between the two groups. 

### Healing status evaluation with MRI

MRI was performed in all patients both preoperatively and 1 year after surgery. Medial meniscal extrusion (MME) was defined as the distance between the medial border of the tibia, excluding any osteophytes, and the medial edge of the MM on the MRI slice displaying the most prominent point of the medial tibial eminence [21]. The change in MME (ΔMME) was calculated by subtracting the preoperative MME value from the postoperative value of MME.

The integrity of the repaired posterior meniscal root was evaluated using MRI at the 1-year follow-up visit. Healing status was classified according to a previously established system as follows: complete healing was defined by continuous meniscal in all three MRI planes (sagittal, coronal, and axial); partial healing was diagnosed when continuity was lost in any one of these planes; and a retear was identified when continuity was absent in all three planes.

### Clinical outcome evaluation

Clinical outcomes were evaluated annually after surgery; however, the present study specifically assessed outcomes preoperatively and at the 2-year postoperative follow-up. The evaluations included the Knee Injury and Osteoarthritis Outcome Score (KOOS), International Knee Documentation Committee (IKDC) subjective knee form, Lysholm knee score, and Tegner activity level scale. Clinical scores within each group were compared between the preoperative and final follow-up assessments. Additionally, the final clinical outcomes were compared between the two groups.

### Statistical analysis

Data are expressed as mean ± standard deviation unless otherwise specified. Statistical significance was defined as *p* < 0.05. The Mann–Whitney *U* test was used to compare the demographic and clinical variables between the AA and PA groups. Categorical variables, including sex and K–L grade, were analyzed using the chi-squared test. All statistical analyses were conducted using SPSS software (version 20.0; IBM, Armonk, NY, USA). All measurements were independently performed by two orthopedic surgeons who were blinded to the surgical procedure. Each measurement was repeated twice at 2-week intervals, and the average was used for analysis. Intraclass correlation coefficients (ICCs) were calculated to determine the intraobserver and interobserver reliability of the variations in the measurements of the distances between the tibial tunnel center and the anatomic center, and width of the MJS. Inter- and intra-observer reliabilities were assessed using the ICC, with values > 0.80 considered reliable. Post hoc power analysis for the postoperative outcome measures (direct tunnel distance on CT and subjective functional IKDC score) was performed using G*Power version 3.1.9.7 (Franz Paul, Kiel, Germany), with effect sizes calculated at a significance level of 0.05. The calculated power ranged from 0.17 to 1.0, based on 54 participants. The power for direct distance measurement on 3D-CT (effect size: 1.38) was 1.0, whereas the statistical powers for the Lysholm score (effect size: 0.37) and subjective functional IKDC score (effect size: 0.19) were 0.38 and 0.17, respectively.

## Results

A total of 77 patients were enrolled in this study. Of these, 23 patients with MMPRTs were excluded according to the predefined exclusion criteria (concomitant high tibial osteotomy and/or cartilage procedure: 12; refusal of CT and/or MRI: 7; follow-up loss: 4). The AA and PA groups included 26 and 28 patients, respectively. No significant differences were found in baseline demographics or preoperative clinical characteristics between the groups (Table 1). The mean age of all patients was 57.0 ± 5.6 years. The mean interval between root repair and postoperative MRI was 13.4 ± 1.7 months (range 12–19 months). Postoperative clinical outcomes were evaluated at 27.8 ± 2.8 months (range 24–35 months). Radiographic reliability was high, with mean inter- and intra-observer ICC values of 0.87 and 0.95, respectively.

**Table 1:** Patient demographics, clinical scores, and radiologic measurements

	Anterior approach group (*n* = 26)	Posterior approach group (*n* = 28)	*p*
Demographics			
Age (years)	58.2 ± 4.1	55.8 ± 6.6	0.145
Sex (male:female)	7:19	5:23	0.423
Body mass index (kg/m^2^)	27.4 ± 4.3	25.8 ± 4.8	0.736
Duration between symptom onset and operation, weeks	6.3 ± 3.8	7.2 ± 4.1	0.386
Preoperative clinical score			
IKDC subjective score	37.8 ± 15.9	39.2 ± 16.8	0.674
Lysholm score	57.3 ± 15.3	59.2 ± 16.2	0.725
Tegner activity scale	2(0–4)	2(0–4)	0.782
KOOS score			
Pain	61.6 ± 16.8	64.8 ± 18.6	0.678
Symptoms	63.7 ± 17.3	61.5 ± 19.4	0.542
ADL	71.6 ± 18.9	73.5 ± 19.8	0.496
Sport/recreation	38.9 ± 23.8	34.7 ± 21.7	0.611
QOL	36.7 ± 18.2	34.6 ± 21.8	0.421
Preoperative radiologic measurements			
Preoperative mechanical axis (varus)	3.1 ± 2.1	3.0 ± 1.9	0.751
Kellgren–Lawrence grade (0/1/2/3/4)	5/15/6/0/0	4/18/6/0/0	0.856
ICRS grade (0/1/2/3/4)	3/5/12/4/2	2/7/12/4/3	0.956
MJS, mm	3.5 ± 1.2	3.5 ± 1.1	0.519
MME, mm	3.2 ± 0.9	2.9 ± 0.9	0.377

Data expressed as mean ± standard deviation or counts

ADL, activities of daily living; IKDC, International Knee Documentation Committee; KOOS, Knee Injury and Osteoarthritis Outcome Score; QOL, quality of life; Sport/Rec, sports and recreational function; MJS, medial joint space; MME, medial meniscus extrusion

Significance differences were assessed using Mann–Whitney U test and Pearson chi-squared test

The anatomic center of the MMPRT footprint was identified on 3D CT at a mean position of 79.4 ± 2.5% posterior and 40.6 ± 2.9% lateral, and the tibial tunnel percentage center of each group is presented in Table 2. In the AA group, the tibial tunnel center was positioned more anteromedially relative to the anatomic center; in the PA group, it was positioned posteromedially. In the AA group, 21 of 26 knees (80.7%) had tibial tunnels located anterior to the anatomic center, and 20 knees (76.9%) were positioned more medially. In the PA group, 15 of 28 knees (53.6%) had tunnels located posterior to the anatomic center, and 24 knees (85.7%) were positioned more medially (Fig. 4).

**Table 2:** Percentage position and absolute distance between the anatomic and tibial tunnel centers at postoperative 1 year

	Anterior approach group (*n* = 26)	Posterior approach group (*n* = 28)	*p*
Absolute distances between anatomic and tibial tunnel centers^a^			
Anteroposterior distance (mm)	3.1 ± 3.3	− 0.5 ± 2.1	< 0.001
Mediolateral distance (mm)	2.3 ± 2.2	2.0 ± 1.4	0.507
Direct distance (mm)	4.9 ± 3.2	2.8 ± 2.0	0.001
Tibial tunnel percentage position^b^			
Anteroposterior position (%)	73.1 ± 2.8	80.5 ± 2.7	
Mediolateral position (%)	37.4 ± 3.1	37.8 ± 3.0	

^a^The absolute distance was recorded as (+) when the tibial tunnel center was located anterior or medial to the anatomic center and (−) when located posterior to the anatomic center

^b^Tibial tunnel percentage position was measured on 3D CT and is expressed as percentages in the anteroposterior and mediolateral directions relative to the tibial plateau, with 0% corresponding to the anterior and medial margins and 100% to the posterior and lateral margins. P values were not calculated for percentage positions.

The mean absolute distances between the tibial tunnel center and the anatomic center were 4.9 ± 3.2 mm in the AA group and 2.8 ± 2.0 mm in the PA group. This distance was significantly smaller in the PA group (*p* = 0.001). Average distances and percentage positions in the anterior, medial, and direct directions are presented in Table 2.

Radiographic analysis revealed significant progression in MJS narrowing and MME from the preoperative to postoperative period. MJS decreased significantly from 3.5 ± 1.2 mm preoperatively to 3.1 ± 1.3 mm postoperatively (*p* = 0.001). MME increased from 3.0 ± 0.9 mm to 4.0 ± 1.6 mm (*p* = 0.022). The mean ΔMJS was 0.4 ± 0.6 mm, and the mean ΔMME was 0.8 ± 1.2 mm. No significant differences in final MJS or MME outcomes were found between the groups (Table 3).

**Table 3:** Comparison of postoperative clinical and radiologic outcomes between the two groups at postoperative 2 years

	Anterior approach group (*n* = 26)	Posterior approach group (*n* = 28)	*p*
Postoperative clinical scores			
IKDC subjective score	68.9 ± 15.8	71.8 ± 14.9	0.455
Lysholm score	81.7 ± 8.2	84.9 ± 9.1	0.282
Tegner activity scale	3 (2–5)	3 (2–5)	0.891
KOOS			
Pain	85.9 ± 8.5	89.6 ± 7.9	0.238
Symptoms	86.7 ± 9.3	88.5 ± 9.4	0.473
ADL	91.6 ± 7.9	90.5 ± 6.8	0.569
Sport/recreation	67.9 ± 21.2	69.1 ± 20.7	0.563
QOL	73.7 ± 18.2	77.1 ± 19.7	0.291
Postoperative radiologic measurements			
Mechanical axis (varus), °	3.3 ± 2.2	3.4 ± 2.3	0.476
MJS, mm	3.2 ± 1.4	3.1 ± 1.2	0.875
Change in MJS (ΔMJS), mm	0.4 ± 0.6	0.4 ± 0.6	0.953
MME, mm	3.6 ± 0.8	4.0 ± 1.6	0.375
Kellgren–Lawrence grade: 0/1/2/3/4	3/13/5/5/0	3/16/7/2/0	0.602
Progression of Kellgren–Lawrence grade, *n* (%)	11	6	0.144
Cartilage grade (modified Outerbridge grade): 0/1/2/3/4	2/4/11/5/4	2/5/13/3/5	0.936
Progression of cartilage grade, *n* (%)	8	5	0.346

MME, medial meniscus extrusion; MJS, medial joint space

**Fig. 5:**
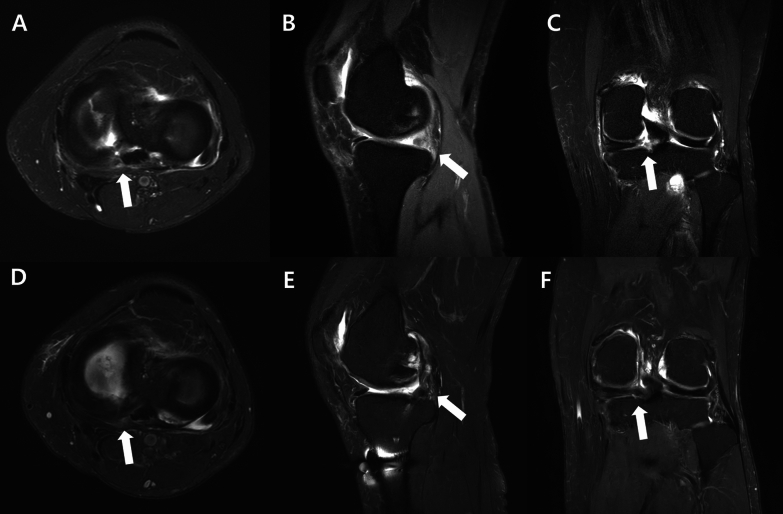
Preoperative and 1-year postoperative MRI scans of a patient who underwent posterior approach repair for a medial meniscus posterior root tear in the left knee. White arrows indicate the tear site in images **A**–**C**, and the healed root in images **D**–**F**. **A** Preoperative axial image; **B** preoperative sagittal image; **C** preoperative coronal image showing the medial meniscus posterior root tear. **D** One-year postoperative axial image; **E** sagittal image; and **F** coronal image demonstrating complete healing with normalization of meniscal signal

On MRI performed at 1 year postoperatively, intact root tissue was observed in 48 patients (88.9%), while healing failure was noted in 6 patients (11.1%). Among those with intact root tissue, 27 patients (50%) showed complete healing and 21 (38.9%) showed partial healing. Healing failure occurred in four patients in the AA group and two patients in the PA group, with no significant difference between the groups (*p* = 0.413) (Fig. 5).

At the 2-year postoperative evaluation, all clinical scores improved significantly compared with baseline. However, no significant differences were observed between the AA and PA groups in the final IKDC, Lysholm, and KOOS scores (Table 3).

## Discussion

This study demonstrated that transtibial pull-out repair of MMPRT in the PA group resulted in significantly more accurate tibial tunnel positioning compared with the AA group. However, although the PA group showed more accurate tibial tunnel placement—as measured by the absolute direct distance from the anatomic center—it did not significantly improve clinical scores or prevent the progression of MJS narrowing compared with the AA group at the final follow-up. Although this finding did not demonstrate a direct advantage in terms of clinical outcomes or medial joint preservation, it highlights the potential technical benefits of the PA in achieving anatomical tunnel placement in MMPRT repairs.

The anatomical positioning of the tibial tunnel has been emphasized in previous studies as a crucial factor in restoring meniscal hoop tension and optimizing joint biomechanics following root repair. Kawada et al. reported that accurate tibial tunnel placement in transtibial pull-out repair for MMPRTs delayed the progression of MJS narrowing and MME, while also contributing to improved clinical outcomes [19]. Similarly, Kamatsuki et al. demonstrated that tibial tunnel positioning closer to the native attachment site significantly improved meniscal healing status and patient-reported outcome measures at 1 year postoperatively [22]. Moreover, Hiranaka et al. [16] found that a tibial tunnel located within 5.8 mm of the native anatomic center was associated with significantly higher meniscal healing rates on second-look arthroscopy, suggesting that anatomical tunnel placement may positively influence the tissue healing.

Our findings indicate that, despite achieving a more accurate tunnel position using the posterior approach, precise placement of the tibial tunnel in both the anteroposterior and absolute dimensions did not significantly affect the progression of MJS narrowing at the final follow-up visit. Furthermore, no significant association was observed between tunnel position accuracy and clinical scores at the final postoperative evaluation. These findings contrast with those of previous reports, which highlighted the clinical and radiological benefits of anatomical tunnel placement.

One possible explanation for this discrepancy is the multifactorial nature of postoperative outcomes following MMPRT repair. Previous studies have identified several patient-related and structural factors, such as advanced age, elevated body mass index, preoperative meniscal extrusion, and varus malalignment. These factors also act as independent predictors of clinical and radiological outcomes following MMPRT repair, regardless of tibial tunnel positioning accuracy [23, 24]. Furthermore, the correction of meniscal extrusion has been recognized as a critical determinant of postoperative healing and symptomatic improvement, in addition to anatomical tunnel positioning [24]. These findings support the notion that, although anatomical tunnel placement may be desirable, its isolated influence on clinical outcomes is likely to be limited when considered within the context of these interacting variables.

In the present study, different suture techniques were employed in the two groups. Owing to the technical limitations of the posterior portal, it was difficult to perform the modified Mason–Allen technique. However, the posterior approach enabled secure fixation by suturing the thick posterior horn tissue. Differences in arthroscopic viewing angles between the anterior and posterior portals may influence tunnel visualization and trajectory, potentially affecting the outcomes observed in this study. Notably, no significant differences in failure or retear rates were found between the groups. These findings suggest that surgical access, anatomical tunnel positioning, and suture configuration may interact in a complex manner. Therefore, future long-term studies should comprehensively evaluate these combined variables. Considering the progressive and degenerative nature of MMPRT, the influence of surgical factors on joint preservation and clinical outcomes should be assessed through extended follow-up. Accurate tibial tunnel placement aligns with the findings from previous biomechanical and radiologic studies, which have emphasized its role in restoring hoop tension and improving the mechanical environment of the knee joint following MMPRT repair. Through finite element analysis, Steineman et al. [25] reported that repairs placed posteriorly or deviating from the anatomical site substantially altered knee joint mechanics, resulting in increased meniscal extrusion and contact pressure. Anterior tunnel placement has been associated with increased tension at the repair site, thereby increasing the risk of suture cut-out or repair failure. Although anatomic placement restored contact mechanics more effectively than anterior or posterior placement, their simulations indicated that the mechanical benefit remained incomplete compared with the intact knee, suggesting the involvement of additional biological factors.

The PA group achieved closer approximation to the anatomic root center; however, the tunnels were positioned slightly posterior to the anatomic root. This may have resulted in a lower-tension environment, potentially reducing the risk of suture cut-out or meniscal retear, but possibly increasing the risk of meniscal extrusion. Consistent with these biomechanical insights, a higher incidence of meniscal retears was observed in the AA group in the present study, potentially due to increased suture tension and limited intraoperative visualization, which may have contributed to suboptimal repair positioning. Although the PA group demonstrated a tendency toward greater meniscal extrusion compared with the AA group, this difference was not significant. Therefore, long-term follow-up studies are warranted to determine the clinical implications of these trends and to further elucidate the relationship between tunnel position and postoperative joint biomechanics.

The PA for MMPRT repair offers distinct technical advantages over anterior approaches, particularly in minimizing soft-tissue disruption and reducing the risk of iatrogenic cartilage damage. Traditional anterior-based transtibial techniques often require the application of valgus stress or partial release of the superficial medial collateral ligament (MCL) to adequately access the posteromedial compartment of the knee. Such maneuvers may increase the risk of MCL-related complication after the surgery. In contrast, the PA enables direct visualization and instrumentation of the root attachment site through a transseptal portal without requiring MCL release. This preserves the integrity of the MCL and avoids additional procedural invasiveness. Furthermore, the constrained MJS encountered during anterior-based tunnel drilling and suture passage can increase the risk of iatrogenic chondral damage, particularly involving the femoral condyle and tibial plateau. The posterior approach may facilitate a more anatomical tunnel position under direct visualization, potentially reducing device misplacement and cartilage injuries. These technical advantages highlight the utility of the posterior approach in improving the safety and precision of MMPRT repairs.

The clinical implications of these findings suggest that anatomical tunnel placement, which can be more consistently achieved with the posterior approach, provides superior positional accuracy. However, given the absence of significant differences in clinical outcomes, this should be interpreted with caution. Attention must also be directed toward biological healing factors, tissue quality, repair tension, and comprehensive postoperative management to optimize the clinical outcomes. These results underscore the multifactorial nature of MMPRT repair outcomes, which rely on a combination of surgical precision, healing response, and patient-specific factors.

This study had some limitations. First, the relatively small sample size may have reduced the statistical power and limited the generalizability of the findings to a broader population. Second, although radiologic parameters such as MJS narrowing and MME were evaluated, second-look arthroscopy was not performed. Consequently, direct visualization of meniscal healing and its correlation with tibial tunnel positioning could not be established. Third, the follow-up duration was only 2 years. Although the AA group had a longer follow-up period, a comparison between the two groups was necessary. The duration may have been insufficient to fully evaluate the long-term progression of osteoarthritis, as degenerative changes may continue beyond the early postoperative period despite initial radiographic stabilization. The modified Mason–Allen technique was not feasible through the posteromedial portal; however, vertical sutures were successfully placed via posteromedial portal, allowing adequate capture of the posterior meniscal tissue. Notably, no inferior meniscal healing rate was observed in the PA group. These limitations should be considered when interpreting the results of this study and underscore the need for larger, long-term prospective studies incorporating both imaging and arthroscopic assessments.

## Conclusions

The PA group achieved more accurate replication of the posterior horn insertion than the AA group; however, no definitive advantages were observed in short-term clinical or radiographic outcomes.

## Data Availability

The datasets analyzed during the current study are available from the corresponding author on reasonable request.

## Abbreviations

ACL: Anterior cruciate ligament
BMI: Body mass index
CT: Computed tomography
ICC: Intraclass correlation coefficient
IKDC: International Knee Documentation Committee
JSW: Joint space width
K–L: Kellgren–Lawrence
KOOS: Knee Injury and Osteoarthritis Outcome Score
MCL: Medial collateral ligament
MJS: Medial joint space
MM: Medial meniscus
MME: Medial meniscus extrusion
MMPRT: Medial meniscal posterior root tear
MRI: Magnetic resonance imaging
OA: Osteoarthritis
PACS: Picture archiving and communication system
PCL: Posterior cruciate ligament
PDS: Polydioxanone suture
PROMs: Patient-reported outcome measures
ΔMME: Change in medial meniscal extrusion
ΔMJS: Change in medial joint space
